# Iron‐Mediated Release of Aged Dissolved Organic Carbon From Waterlogged Peatland Under Warming

**DOI:** 10.1002/advs.202522234

**Published:** 2026-07-23

**Authors:** Guohua Dai, Zhiying Yang, Zongguang Liu, Wanjia Hu, Lixiao Ma, Enze Kang, Wanqing Luo, Yunpeng Zhao, Chengzhu Liu, Juan Jia, Ting Liu, Chen He, Quan Shi, Jianliang Liu, Yongheng Gao, Huai Chen, Hailong Zhang, Meixun Zhao, Xiaojuan Feng

**Affiliations:** ^1^ State Key Laboratory of Forage Breeding‐by‐Design and Utilization and Key Laboratory of Vegetation and Environmental Change Institute of Botany Chinese Academy of Sciences Beijing China; ^2^ China National Botanical Garden Beijing China; ^3^ University of Chinese Academy of Sciences Beijing China; ^4^ State Key Laboratory of Heavy Oil Processing China University of Petroleum Beijing China; ^5^ Mountain Ecological Restoration and Biodiversity Conservation Key Laboratory of Sichuan Province Chengdu Institute of Biology Chinese Academy of Sciences Chengdu China; ^6^ Zoige Wetland Ecology Research Station Chengdu Institute of Biology Chinese Academy of Sciences Hongyuan China; ^7^ Aba Teachers College Aba Tibetan and Qiang Autonomous Prefecture China; ^8^ Laoshan Laboratory Qingdao China

**Keywords:** dissolved organic carbon, iron reduction, radiocarbon, warming, Zoige peatland

## Abstract

Peatlands contribute ∼20% of dissolved organic carbon (DOC) exported from land to sea. However, potential changes in DOC release from waterlogged peatlands under warming remain poorly understood. Here we conduct an in situ warming experiment in the waterlogged high‐altitude peatlands (Zoige), integrating five years of field manipulations with monthly/quarterly porewater monitoring by >100 ^14^C measurements, organic matter molecular characterization, and microbial community profiling. We show that warming generally increases peatland porewater DOC concentrations, albeit via different mechanisms under contrasting hydrological regimes. In drained peatlands, increased DOC, characterized by modern carbon with high aromaticity, is linked to elevated plant inputs under warming. Conversely, in waterlogged peatlands, warming releases aged (∼890 years before present; 95% credible interval: 283–1935 years), lipid‐rich DOC through enhanced iron reduction by increased iron‐reducing bacteria. Our findings highlight that warming destabilizes aged carbon via enhanced iron reduction from waterlogged alpine peatlands—a previously overlooked aquatic pathway generating positive climate feedback in these globally significant ecosystems.

## Graphical abstract

This study conducts a five‐year in situ warming experiment in high‐altitude peatlands to examine dissolved organic carbon (DOC) release. The study shows that warming promotes the release of plant‐derived modern DOC in drained peatlands, but amplifies aquatic export of century‐old DOC from waterlogged peatlands via Fe‐mediated DOC mobilization—a potential and previously overlooked pathway for positive climate feedback.

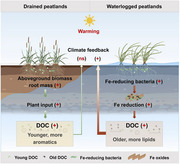

## 1 Introduction

Peatlands store approximately one third of the global soil carbon (ca. 650 Gt C) [1, 2, 3] and play a key role in atmospheric CO_2_ drawdown and climate change mitigation [2, 4]. Dissolved organic carbon (DOC) export represents a major pathway of carbon loss from peatlands, constituting up to 50% of net ecosystem CO_2_ exchange therein [5, 6]. Moreover, peatlands export more DOC than any other terrestrial ecosystem, accounting for 20% of the DOC ultimately transferred from land to sea [7]. Hence, DOC release from peatlands has vital impacts on the regional carbon budget, water quality, and biogeochemical cycling in the watersheds [8, 9]. However, the response of peatland DOC export to climate warming remains poorly constrained, hampering accurate predictions of peatland carbon‐climate feedbacks and watershed biogeochemical processes in a changing climate [10, 11].

A number of warming experiments conducted in upland ecosystems and drained peatlands (defined here as having a water table > 10 cm below the surface) have documented a general increase in soil DOC concentrations after warming [12, 13]. This phenomenon is primarily attributed to stimulated plant productivity and elevated inputs of recent photosynthates as dissolved organic matter (DOM) [13, 14], despite potentially enhanced mineralization by microbes [15]. By comparison, DOC responses to warming in waterlogged peatlands are under‐investigated, partly due to experimental challenges associated with manipulations in ecosystems with standing and free‐flowing water. The few existing studies report contrasting results, ranging from decreased [16], unchanged [17], to increased [18] DOC concentrations in waterlogged peatlands under warming. Given the wide coverage (80%–85% of global peatlands) and tremendous carbon storage (∼530 Gt C) of waterlogged peatlands [19], it is essential to reveal the response and the underlying mechanisms of DOC to warming therein to better predict future peatland DOC dynamics and to inform conservation strategies for these critical ecosystems.

Waterlogged peatlands may show distinct DOC responses to warming compared with uplands and drained peatlands. For instance, warming may suppress the growth of moisture‐sensitive plants in peatlands (e.g., *Sphagnum*) [20] due to soil desiccation and enhance microbial decomposition [16], thereby decreasing DOC concentrations. Alternatively, warming may accelerate microbially driven iron (Fe) reduction [21, 22, 23] by increasing the activity and population of Fe‐reducing bacteria [24], which plays a key role in DOM release under waterlogged, anoxic/hypoxic conditions [18, 25, 26]. Importantly, over 20% of soil organic carbon (SOC) is stored as Fe‐bound organic carbon (OC) in peatlands [27, 28, 29], which exhibits slower turnover than bulk SOC [30]. Therefore, the potential release of this slow‐cycling OC pool from waterlogged peatlands may trigger a positive climate feedback [31, 32], in contrast to drained peatlands and uplands, where warming‐increased DOC primarily originates from recent photosynthates [12, 13, 33]. However, field evidence for aged DOC release from waterlogged peatlands in association with Fe reduction is currently lacking.

The Zoige peatlands, the world's largest alpine peatland complex on the Qinghai‐Tibetan Plateau, harbor the headwaters of major Asian rivers (e.g., the Yellow, Yangtze and Mekong Rivers) and are experiencing a faster‐than‐average warming trend [34]. Yet, warming effects on DOC dynamics and export in the Zoige peatlands have not been explicitly explored in the field. Since the 1970s, regional drainage has lowered water tables (>15 cm below surface) in ∼41% of the Zoige peatlands [35], creating mosaic landscapes and a unique opportunity to compare warming impacts on DOC release in peatlands under different moisture regimes.

Here, we conducted a long‐term field warming experiment in waterlogged peatlands of Zoige using open‐top chambers (OTCs) to investigate peatland DOC responses to warming, in comparison to drained peatlands nearby. To allow effective manifestation of the warming effect, we installed two intersecting stainless steel barriers extending 75 cm belowground around each experimental plot to minimize (but not completely eliminate) DOM exchange with non‐manipulated peat. Soil porewater DOC concentrations were monitored at three depths (10, 30, and 50 cm) during 14 sampling campaigns (at monthly or quarterly frequency) over five consecutive growing seasons (2020–2024). To distinguish DOM sourcing from recent plants vs. aged soil components (potentially Fe‐bound moieties), we examined DOC radiocarbon (^14^C) content and DOM molecular composition using ultrahigh‐resolution mass spectrometry. To elucidate mechanisms regulating DOC variations under warming, we measured plant productivity, soil redox potential, microbial biomass, and soil enzyme activities. In particular, to reveal the role of microbially driven Fe reduction in DOC mobilization, we quantified ferrous Fe (Fe^2+^) concentrations in porewater as well as hydrochloric acid (HCl)‐extractable ferrous Fe [Fe(II)_HCl_] and Fe‐reducing bacteria abundance in soils. With these comprehensive approaches, we aimed to test the hypothesis that warming promoted aged DOC release in waterlogged peatlands through microbially driven Fe reduction, in contrast to drained peatlands where warming primarily elevated DOC due to increased recent plant inputs. Elevated DOC release from waterlogged peatlands may hence pose a strong positive climate feedback and deserves close attention.

## 2 Results and Discussion

### 2.1 Warming Increases Peatland Porewater DOC

**FIGURE 1:**
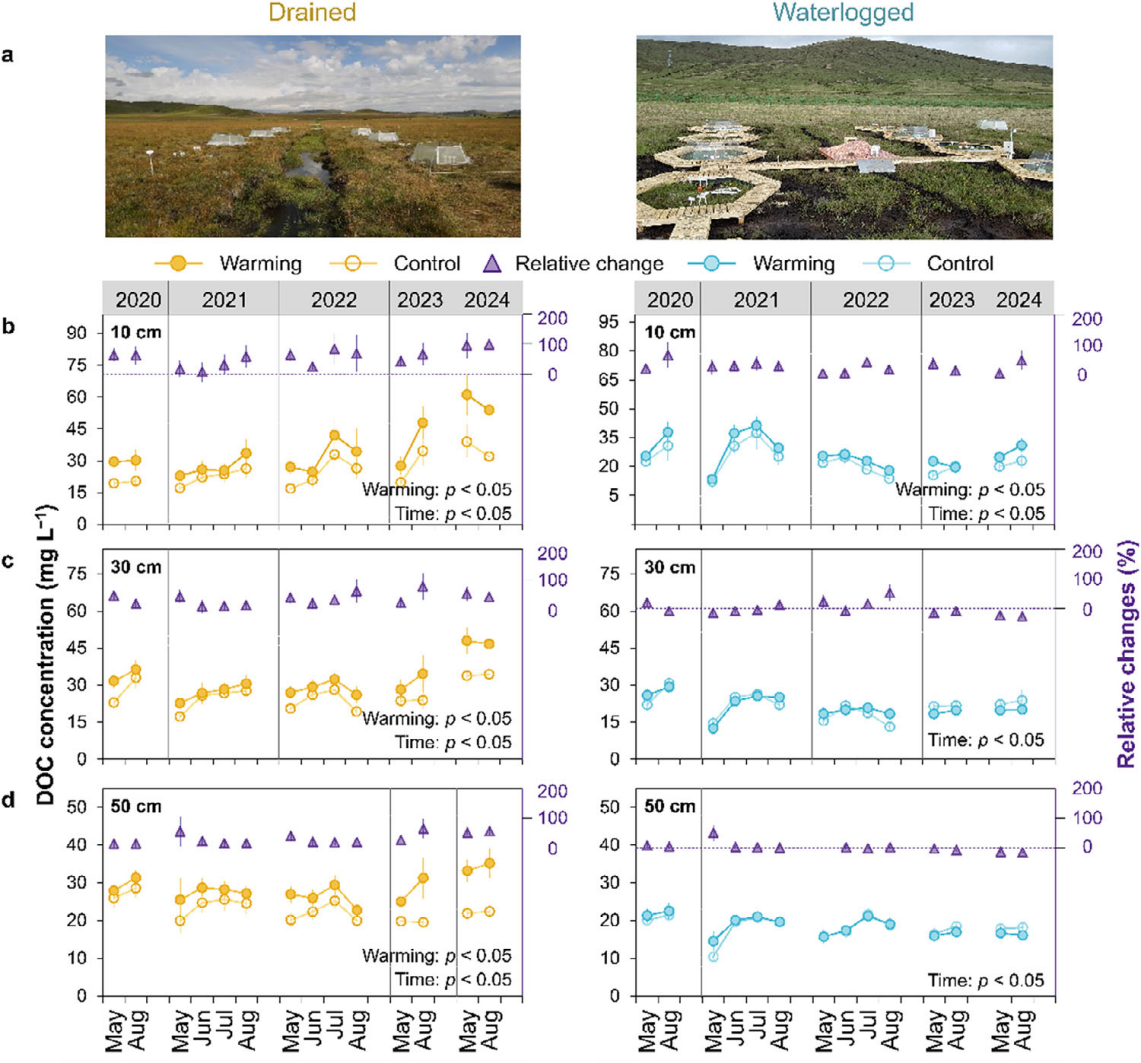
Effects of warming on porewater Dissolved Organic Carbon (DOC) concentrations in zoige peatlands over five consecutive growing seasons (2020–2024). (a) Field photographs of the warming experiment, (b) 10 cm Depth, (c) 30 cm Depth, and (d) 50 cm Depth. Percentage changes (purple triangle) are calculated as the concentration offset between the warmed and control plots relative to that in the control plots of the respective sampling time. Error bars are standard errors of the mean values (*n* = 6).

Our field warming experiment encompassed two adjacent sites (∼150 m apart) at an elevation of 3400 m (Figure S1). The waterlogged site with water tables within ± 5cm of the soil surface in the growing season was dominated by sedge (mainly *Carex muliensis*, *Schoenoplectus triqueter* and *Blysmus sinocompressus*). The drained site had water tables of 34 ± 2 cm (mean ± SE, *n* = 96) below the surface in the growing season (Figure S2) and was dominated by grasses (mainly *Deschampsia cespitosa*, *Elymus dahuricus* and *Argentina anserine*). We established twelve 2 m × 2 m plots (spaced 2 m apart) at each site in the summer of 2019 (Figure 1a). The OTC treatment, randomly applied to half of the plots (six replicates for each treatment) in September 2019, increased the annual mean air temperature by 1.38°C (in 2020–2024) at 30 cm above the peat surface (Figure S1), effectively simulating current climate warming trends projected by the Intergovernmental Panel on Climate Change [36]. Soil temperature increases were smaller in magnitude than air warming, averaging 0.56°C and 0.08°C at 10 cm, 0.33°C and 0.20°C at 30 cm, and 0.41°C and 0.28°C at 50 cm depth for the waterlogged and drained sites, respectively (Figure S3). Notably, OTC‐induced soil warming was greater in winter than in summer (Figure S3), which mirrors the asymmetric seasonal warming pattern expected under real climate change scenarios (greater winter warming) [36]. This suggests that our OTC warming experiment provides a realistic belowground thermal regime for assessing soil carbon responses to future climate warming.

**FIGURE 2:**
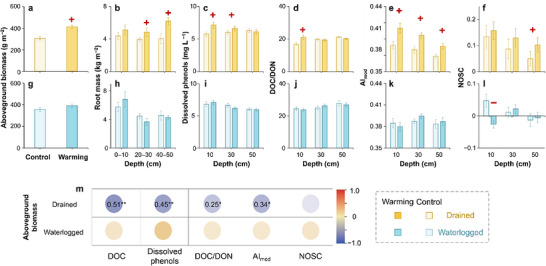
Effects of warming on plants and porewater Dissolved Organic Carbon (DOC) chemistry, and their relationships at the drained (a–f) and waterlogged sites (g–l). (a, g) Mean aboveground biomass (2020–2024; *n* = 30); (b, h) Mean root mass after two (2021) and four (2023) years of warming treatment (*n* = 12); (c, i) Mean dissolved phenol concentrations (2021–2024; *n* = 66); (d, j) Mean ratio of DOC to Dissolved Organic Nitrogen (DOC/DON) (2020–2024; *n* = 72); (e, k, f, l) Mean modified aromaticity index (AI_mod_) and Nominal Oxidation State of Carbon (NOSC) from early (May) and peak (August) growing seasons in 2020–2022 (*n* = 6), respectively. + and − show significant increase and decrease (*p* < 0.05), respectively. (m) Spearman's rank correlation coefficients (R) between aboveground biomass and DOC concentration and chemical composition. The color scale indicates the strength and sign of the correlations. *p  <  0.05; **p  <  0.01.

For five consecutive growing seasons after the experiment started, warming significantly increased soil porewater DOC concentrations during our 14 sampling campaigns by 57 ± 10% (*n* = 84), 35 ± 7% (*n* = 84) and 31 ± 6% (*n* = 84) at 10, 30 and 50 cm depth at the drained site, respectively (*p* < 0.05; Figure 1b–d). The DOC increase coincided with increased plant aboveground biomass (by 36 ± 9%; *n* = 30; *p* < 0.05; Figure 2a; Figure S4a) and root mass, particularly at 40–50 cm (51 ± 19%; *n* = 12; *p* < 0.05; Figure 2b; Figure S4b). A strong positive correlation between surface (10 cm) DOC and aboveground biomass (*r* = 0.51; *p* < 0.05; Figure 2m) implied that increased plant inputs were likely a key driver of DOC increases in the drained peatlands, consistent with previous studies [13, 33]. This conclusion was supported by further analysis of DOM composition, as warming significantly increased indicators of plant inputs [13, 37, 38], including dissolved phenols [38], ratio of DOC to dissolved organic nitrogen (DOC/DON) [12] and DOM aromaticity index (AI_mod_, assessed by Fourier transform ion cyclotron resonance mass spectrometry, FT‐ICR MS; all *p* < 0.05; Figure 2c–e) [39]. A positive correlation was also found between aboveground biomass and the above indicators (*p* < 0.05; Figure 2m). By comparison, no significant difference was observed in soil microbial biomass, enzyme activities or microbe‐derived DOM moieties (such as aliphatic compounds based on FT‐ICR MS) [40] between the warmed and control treatments (*p* > 0.05; Figure S5a), implying that the observed increase in plant inputs did not lead to a detectable enhancement of microbial biomass or activity, and microbial contribution to DOC increases was minimal. Hence, warming‐induced increase in DOC concentrations was primarily driven by elevated plant inputs at the drained site.

By contrast, porewater DOC concentrations only increased at the surface (i.e., 10 cm) of the waterlogged peatlands after warming (by 26 ± 6%; *n* = 84; *p* < 0.05; Figure 1b–d), while plant aboveground biomass, root mass and DOM indicators of plant inputs (e.g., dissolved phenols, DOC/DON, or AI_mod_) remained unchanged after warming relative to the control (*p* > 0.05; Figure 2g–k; Figure S4c,d). Moreover, neither surface DOC concentrations nor any of the aforementioned DOM indicators showed any significant correlation with aboveground biomass (*p* > 0.05; Figure 2m). These results collectively suggested that plant input was not the cause of increased surface DOC. Utilizing chloride (Cl^−^) as a conservative tracer to assess the effect of evapotranspiration on DOC concentrations [33, 38], we found that warming did not alter the ratio of DOC to Cl^−^ (DOC/Cl^−^; *p* > 0.05; Figure S6). Hence, evapotranspiration (if ever enhanced) did not significantly concentrate DOC in the warming treatment, either. Additionally, hydrological redistribution (e.g., lateral flow or vertical upward transport) is also unlikely to explain the observed surface DOC increase, as water‐table levels were comparable between treatments (*p* > 0.05; Figure S2) and the warming effect was limited to the surface layer only. Instead, the nominal oxidation state of carbon (NOSC) significantly decreased in the DOM at 10 cm (*p* < 0.05; Figure 2l), reflecting an increase of reduced, lipid‐like compounds (characterized by low NOSC) [41]. The lipid‐like compounds were previously shown to be released through reductive dissolution of Fe‐bearing minerals during permafrost thaw [42, 43], likely due to their weak binding affinity for Fe minerals compared to aromatic constituents [44, 45]. These results therefore suggested that the potential release of Fe‐bound organic moieties may have contributed to the observed DOC increase at the surface of the waterlogged peatlands.

### 2.2 Warming Promotes Fe Reduction in Waterlogged Peatlands

**FIGURE 3:**
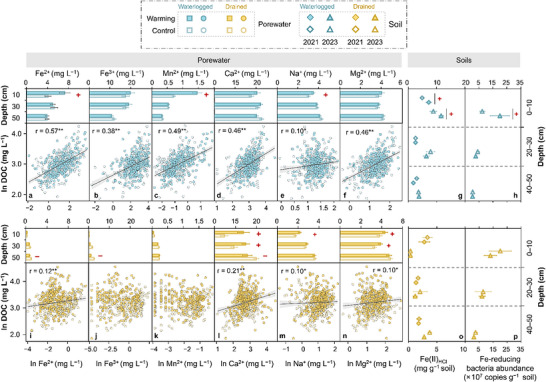
Effects of warming on iron (Fe) species and major cations in porewater, hydrochloric acid (HCl)‐extractable ferrous Fe [Fe(II)_HCl_] and Fe‐reducing bacteria abundance in soils, and their relationships with dissolved organic carbon (DOC). (a–h) Waterlogged site; (i–p) Drained site. (a–f, i–n) Porewater concentrations of ferrous Fe (Fe^2+^), ferric Fe (Fe^3+^), manganese (Mn^2+^), calcium (Ca^2+^), sodium (Na^+^), and magnesium (Mg^2+^), and their correlations with DOC; (g, o) Soil Fe(II)_HCl_ contents; (h, p) Fe‐reducing bacteria abundance in soils. + and − show significant increase and decrease due to warming (*p* < 0.05), respectively. Error bars are standard errors of the mean values (*n* = 84 for all cations in (a–f) and (i–n); *n* = 6 in (g, h) and (o, p)). ^*^ *p*  <  0.05; ^**^ *p*  <  0.01.

To confirm that warming enhanced DOM release via Fe reduction in the waterlogged peatlands, we quantified Fe^2+^ and other major cations in soil porewater, including ferric Fe (Fe^3+^), manganese (Mn^2+^), calcium (Ca^2+^), sodium (Na^+^), and magnesium (Mg^2+^) (Figure 3). Five years (2020–2024) of porewater monitoring showed that warming doubled Fe^2+^ and Mn^2+^ concentrations at 10 cm (*p* < 0.05), while the other cations (with a slight increase in Na^+^) remained unaffected (*p* > 0.05; Figure 3a–f). Moreover, Fe^2+^ showed the strongest positive correlation with DOC among all examined cations (*r* = 0.57; *p* < 0.05; Figure 3a–f), providing diagnostic evidence that Fe^2+^ release from Fe reduction was linked to DOC increases in the waterlogged peatlands [18], because if physical desorption were the dominant mechanism, DOC would likely correlate similarly with other cations (e.g., Ca^2+^). This interpretation was reinforced by a parallel increase in Fe(II)_HCl_ contents (*p* < 0.05; Figure 3g) and decreased redox potentials (*p* < 0.05; Figure S7) in the surface soils (0–10 cm) of waterlogged peatlands under warming, pointing to enhanced Fe reduction [18, 46].

Given that Fe reduction is primarily driven by Fe‐reducing bacteria in natural habitats [47, 48], we identified putative Fe‐reducing bacteria at the genus level and quantified their relative abundance through 16S rRNA gene sequencing after four years of field warming (in August 2023). As expected, warming significantly increased the abundance of putative Fe‐reducing bacteria (e.g., *Bacillus*, *Anaeromyxobacter*, *Geobacter*) [49] in the waterlogged surface soils relative to the control (0–10 cm; *p* < 0.05; Figure 3h). The response of Fe‐reducing bacteria to warming was depth dependent, with no changes detected below 10 cm, likely due to (1) limited thermal transfer of OTC warming to deeper soils, as OTCs passively raise air temperature and the warming signal typically attenuates with soil depth [50], and (2) a lower abundance of Fe‐reducing bacteria at depth (e.g., 7.0 × 10^8^ vs. 1.7 × 10^7^ copies g^−1^ dry soil at 0–10 vs. 40–50 cm; Figure 3h). This result explained the depth‐constrained response of Fe reduction and DOC increase to warming (both limited to the surface 10 cm), with lateral exchange playing a negligible role due to very low hydraulic conductivity at depth [51].

In contrast to the waterlogged peatlands, the drained peatlands exhibited no significant changes in Fe‐reducing bacteria under warming (*p* > 0.05; Figure 3p). Notably, the drained peatlands had much lower porewater Fe^2+^ concentrations (0.62 ± 0.04 mg L^−1^) and soil Fe(II)_HCl_ contents (1.78 ± 0.12 mg g^−1^ dry soil) than the waterlogged counterparts (4.95 ± 0.25 mg L^−1^ and 5.30 ± 0.31 mg g^−1^ dry soil, respectively; Figure 3i,o), while neither changed after warming (*p* > 0.05). Unlike the waterlogged peatlands, Fe^2+^ did not show any stronger correlations with DOC than other major cations in the porewater of the drained peatlands (*r* = 0.10–0.21; Figure 3i–n). Collectively, these results confirmed that warming promoted microbially driven Fe reduction, thereby driving DOC release in surface soils of the waterlogged but not drained peatlands.

### 2.3 Aged DOC Release From Warmed Waterlogged Peatlands

**FIGURE 4:**
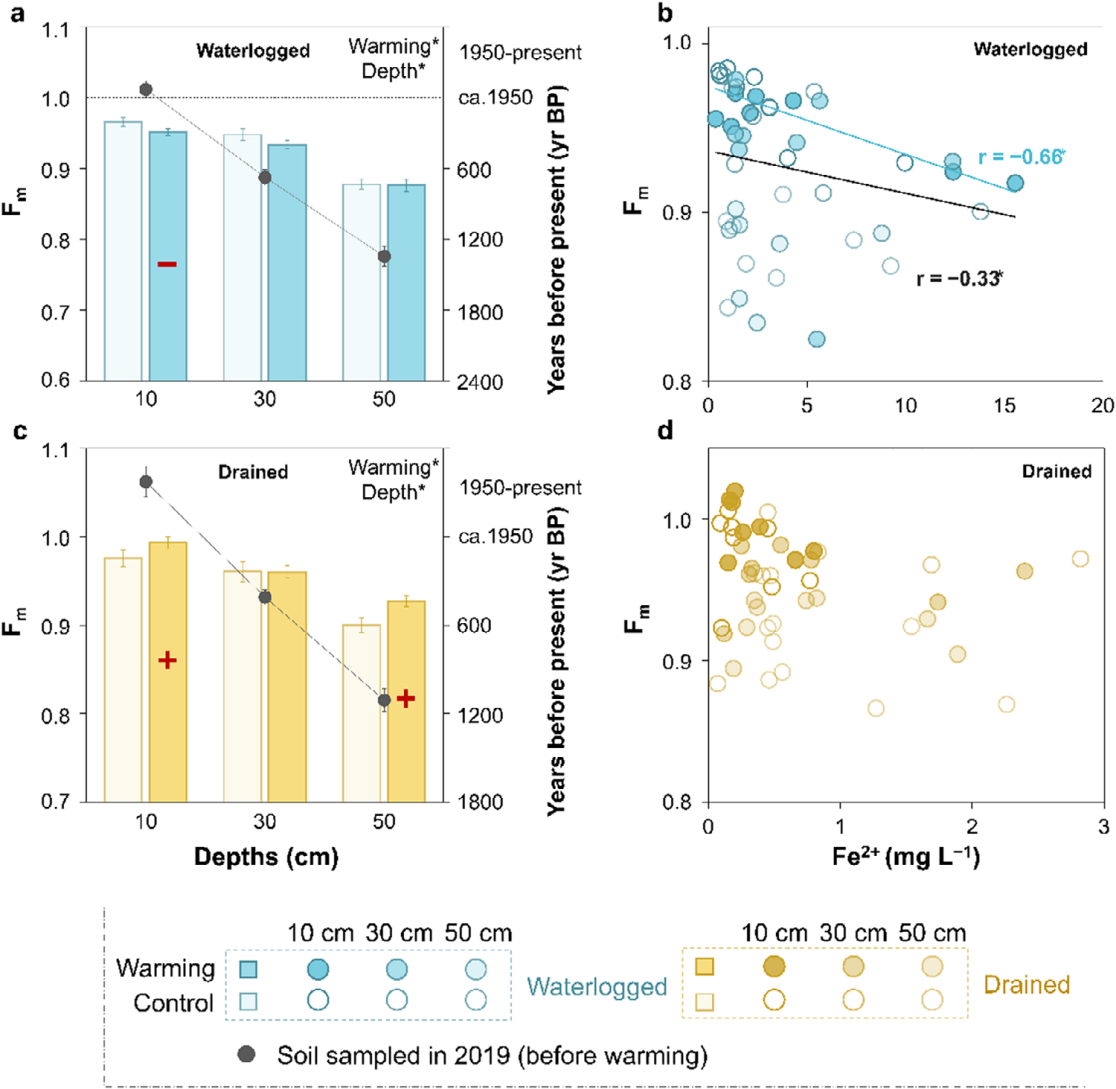
Changes in ^14^C of dissolved organic carbon (DOC) due to warming, and relationships between DO^14^C and Fe^2+^ at waterlogged (a, b) and drained sites (c, d). The ^14^C content is expressed as a fraction modern (F_m_) and the conventional radiocarbon age in years before present (Yr BP); (a, c) ^14^C values; (b, d) relationships between F_m_ and Fe^2+^. + and − show significant increase and decrease (*p* < 0.05), respectively. Error bars are standard errors of the mean values (*n* = 8 for DOC, except *n* = 7 at 50 cm in warmed plots at the waterlogged site; *n* = 3 for bulk SOC). Blue and black lines represent significant correlations (*p* < 0.05) at 10 cm (*n* = 16) and across the entire soil profile (*n* = 47) at the waterlogged site, respectively. ^*^ *p*  <  0.05.

Given that Fe‐bound OC is generally older than bulk SOC in wetlands [30], its release under warming may change the ^14^C content of DOC in peatlands. To test this hypothesis, we measured ^14^C of DOC samples collected from both early (May) and peak (August) growing seasons of four sampling years (2020–2022 and 2024). At the waterlogged site, warming significantly reduced surface porewater DOC ^14^C content (at 10 cm), with fraction modern (F_m_) decreasing from 0.966 ± 0.008 (equivalent to a conventional ^14^C age of 277 ± 52 yr before present, yr BP; *n* = 8) [52] to 0.952 ± 0.007 (398 ± 57 yr BP; *n* = 8; *p* < 0.05; Figure 4a). Furthermore, DOC‐F_m_ showed a strong negative correlation with porewater Fe^2+^ both at 10 cm (r = −0.66; *n* = 16; *p* < 0.05) and across the entire soil profile (r = −0.33; *n* = 47; *p* < 0.05; Figure 4b), supporting our hypothesis that Fe reduction released aged DOC into the waterlogged peatlands.

Assuming that the background (non‐warmed) DOC had the same F_m_ value as in the control, we applied a Bayesian hierarchical mixing model to derive F_m_ values of 0.895 (95% credible interval: 0.786–0.965) for the released DOC from Fe‐bound moieties (details in Methods). These F_m_ values corresponded to conventional ^14^C ages of ∼890 yr BP (95% credible interval: 283–1935 yr BP), much older than bulk SOC (F_m_ = 1.012 ± 0.012, indicating modern carbon; *n* = 3; Figure 4a) at the same depth (0–10 cm) before warming (August 2019). This result again highlighted that the warming‐released DOC was sourced from aged Fe‐bound OC. Consistent with depth‐constrained Fe reduction (Figure 3), no warming effect was detected on DOC‐F_m_ at depths (30 and 50 cm) (*p* > 0.05; Figure 4a).

In contrast, at the drained site, warming significantly increased F_m_ of porewater DOC at both 10 cm (0.994 ± 0.007 in the warmed vs. 0.976 ± 0.010 in the control plots; *n* = 8; *p* < 0.05; Figure 4c) and 50 cm (0.928 ± 0.006 vs. 0.900 ± 0.008; *n* = 8; *p* < 0.05). In addition, DOC‐F_m_ showed no correlation with porewater Fe^2+^ (*p* > 0.05; Figure 4d). These results confirmed that DOC increases in the warmed drained peatlands were primarily driven by contemporary plant inputs.

## 3 Implications

**FIGURE 5:**
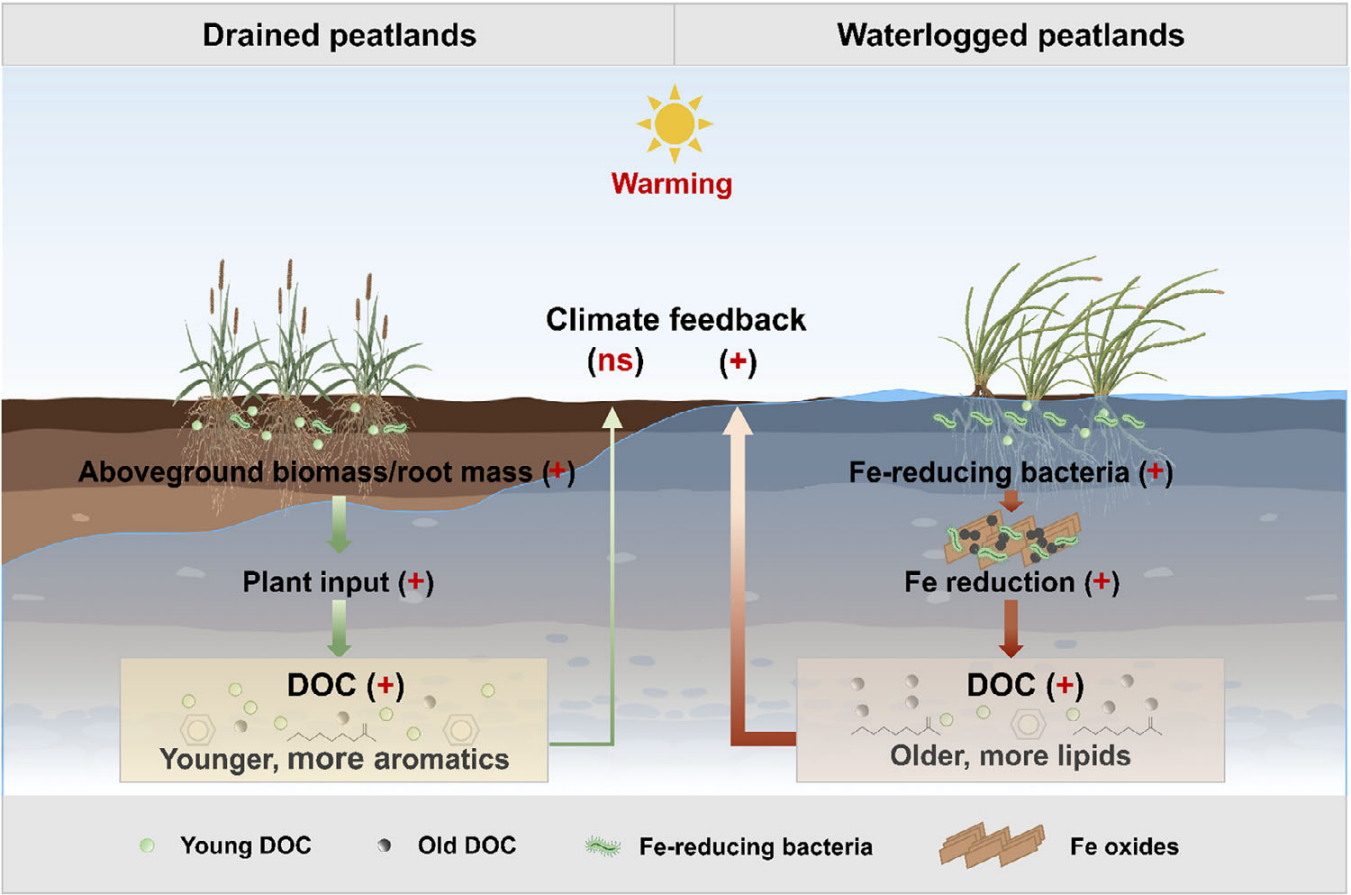
Conceptual figure contrasting mechanisms regulating dissolved organic carbon (DOC) dynamics in response to warming in drained versus waterlogged peatlands. Under warming, drained peatlands release modern, aromatic‐rich DOC derived from increased plant inputs. In contrast, waterlogged peatlands mobilize aged, more lipid‐like DOC through microbially driven iron reduction.

Employing an in situ manipulation experiment in the waterlogged high‐altitude peatland of Zoige, our study, featuring monthly to quarterly monitoring over five consecutive growing seasons, uncovered an overall DOC increase in the waterlogged and drained peatlands under warming, albeit with fundamentally distinct mechanisms and potentials for climate feedback. Drained peatlands released modern, aromatic‐rich DOC derived from increased plant inputs under warming (Figure 5). In contrast, waterlogged peatlands released aged DOC with more lipid‐like moieties due to increases in microbially driven Fe reduction under warming (Figure 5). Critically, the aged DOC released via Fe reduction is originally protected by Fe oxides with a long residence time, characterized by low aromaticity, and potentially prone to microbial mineralization upon its entry into the aquatic downstream [7, 43, 53]. Given that 45%–69% of DOC entering inland waters is typically mineralized during fluvial transport [53, 54], the mobilization of this aged and labile DOC under warming hence represents a previously overlooked pathway for the century‐old carbon to enter into the atmosphere via aquatic systems. This process has the potential to trigger positive carbon‐climate feedbacks (Figure 5) [31, 32], although this mechanism's applicability requires further verification, and the exact fate of the exported DOC (mineralization vs. burial) remains to be quantified. This contrasts sharply with drained peatlands, where the increased modern DOC mainly stems from atmospheric CO_2_ fixed within the recent growing seasons, thereby contributing negligibly to the carbon‐climate feedback.

Admittedly, this study provides an initial estimate based on a warming experiment in Zoige. Notably, our results showing that warming‐induced Fe reduction and aged DOC release were limited to the surface 10 cm came from an OTC warming experiment, which has limited thermal penetration into deeper profiles and thus cannot represent the response of deeper peat layers where the majority of carbon is stored. Future whole‐profile warming experiments are needed to verify whether this mechanism operates at depth. Additionally, the magnitude of warming‐induced aged DOC export via Fe reduction is influenced by multiple factors, such as SOC stock and age, redox potential changes, abundance of Fe‐reducing bacteria, hydrology, and peat structure, etc [32, 55]. While our results demonstrate Fe reduction as the key mechanism driving aged DOC release in Zoige, this phenomenon needs to be confirmed across other peatland types, such as boreal and tropical systems with different hydrogeochemical conditions and microbial functional guilds (e.g., composition and abundance of Fe‐reducing bacteria) [4, 24]. Nevertheless, our study highlights a fundamental dichotomy in climate feedback potentials between drained and waterlogged peatlands, while current understanding of peatland DOC dynamics under warming relies heavily on studies in drained peatlands. We emphasize that aged DOC export via enhanced Fe reduction is a so‐far overlooked pathway for accelerated peat carbon destabilization under warming. Integrating Fe‐mediated peatland carbon cycling into Earth system models will improve predictions of carbon‐climate feedbacks in these extensive, carbon‐rich ecosystems.

## 4 Experimental Section

### 4.1 Site Description

The field warming experiment was situated in the Zoige peatlands (∼3400 m elevation), which cover 4605 km^2^ on the northeastern margin of the Qinghai‐Tibetan Plateau. Mean annual temperature is 1.4°C and mean annual precipitation is ∼800 mm, with approximately 80% of it occurring during the growing season (May–September). From 1961 to 2020, temperatures on the Plateau have climbed steadily at a rate of 0.35°C every 10 years, more than twice the global average, while annual precipitation has risen by an average of 7.9 mm every 10 years (https://www.cma.gov.cn).

In a pristine or intact state, Zoige peatlands are generally characterized by a seasonally fluctuating water‐table level with surface water (waterlogging) present in summer [56]. However, at the beginning of the 1970s, large‐scale drainage of peatlands was conducted to increase grain and livestock production. Long‐term drainage (>50 years) has caused a lowering of the water table by >15 cm and a significant alteration to the plant community [57]. Nearly 1000 artificial drainage channels with a total length of 2864 km have drained nearly 41% of the total area of the Zoige peatland [35]. Therefore, the Zoige peatlands consist of two distinct sub‐habitats: (1) drained peatlands with the water‐table level mostly below 10 cm in summer, dominated by grasses (mainly *Deschampsia cespitosa*, *Elymus dahuricus* and *Argentina anserine*); (2) waterlogged pristine peatlands with water‐table level within ±5 cm of the soil surface most of the year, dominated by sedge (mainly *Carex muliensis*, *Schoenoplectus triqueter* and *Blysmus sinocompressus*). To explore how warming‐induced DOC release differs in contrasting water‐table regimes, we established a field warming experiment at both drained and waterlogged sites.

### 4.2 Experimental Design

The field warming experiment was established in August 2019 in a fenced 200 m × 300 m area of Ruokeba (33°4′5″ N, 102°33′52″ E, 3467 m elevation), one of the largest fens in Zoige peatlands. A long‐term drainage ditch (constructed in the 1970s; ∼1 km long, 2‐m wide and 1‐m deep) went through the fenced area and drained into a small river. Twelve 2 m × 2 m plots (spaced 2 m apart) were set along each side of the drainage ditch at the drained site (with water tables of 34 ± 2 cm; Figure 1a; Figure S1). Similarly, twelve plots were established at the adjacent waterlogged pristine site (150 m away from the drainage ditch), which has a water‐table of 2 ± 2 cm relative to the soil surface (Figure S2) and shares similar topography, geological setting, and peat properties (including peat depth, origin and degree of humification) with the drained site (Figures S1, S8, and S9). To prevent rapid active DOC exchange with the surrounding peat and optimize warming within the plots, each plot was enclosed by two intersecting stainless steel barriers extending 75 cm below and 25 cm above the soil surface in this study, although we acknowledge the potential hydrological influence of the barriers. Baseline soil samples were collected from both drained and waterlogged sites (3 replicates per site) in August 2019 prior to warming initiation for soil water content, bulk density, pH, SOC, SOC to total nitrogen (TN) ratio (SOC/TN), SOC‐^14^C, and the degree of humification analysis (detailed sampling protocols are provided below). Six plots at each site were randomly assigned to control or warming treatments. Elevated boardwalks and planks were built to allow access to all plots at the waterlogged site. Hexagonal OTCs were applied to achieve passive warming, which are used by the International Tundra Experiment (https://www.gvsu.edu/itex/) to simulate climate warming in remote areas [50]. The OTC (60° inclination of the panels, 60 cm tall, and 64 and 98 cm top and basal diameter, respectively) was made of transparent polymethyl methacrylate and placed in the field to achieve year‐round warming. Air temperature (monitored from September 2020) at 30 cm above the soil surface and soil temperature (from September 2019) at 10, 30 and 50 cm depths have been monitored at 30‐min intervals using EM50 data loggers (Decagon Devices, Pullman, WA, USA) in selected plots: 2 warming and 1 control in the drained regime; 2 warming and 2 control in the waterlogged regime.

### 4.3 Plant Survey and Soil Physicochemical Analyses

To examine variations in plant inputs, we determined the aboveground biomass and root mass at the peak growing season. Specifically, at the end of August every year, aboveground biomass was harvested at each plot from one (2019–2022), two (2024), or three (2023) random subplots (50 × 50 cm) to the ground level, and oven‐dried at 65°C to a constant weight for measurement. Root mass was measured by handpicking all visible plant roots out of a known volume of freeze‐dried soil sample (detailed below). Dead roots were not separated due to difficulties in distinguishing living roots among the massive volume of dead ones.

After removal of aboveground biomass, soil samples were collected from two soil cores per plot at a depth of 60 cm (and to 100 cm in 2019) using a specialized corer (diameter of 5 cm) to minimize compaction at the end of August in 2019 (before warming; only 3 replicates were sampled per treatment), 2021, and 2023. Soil cores were sliced at 10 cm intervals in the field and transported (cooled by ice bags) to the laboratory immediately. Notably, soil water content and bulk density were analyzed across the soil profiles (0–100 cm) collected in 2019. For other properties (e.g., pH, SOC), soil samples from the 0–10, 20–30 and 40–50 cm layers were analyzed in this study. Approximately 2 g of the fresh soil was stored at −80°C for microbial DNA extraction, and ∼5 g of the fresh soil was immediately analyzed for extracellular enzyme activity, Fe(II)_HCl,_ and moisture. The remaining soil samples were freeze‐dried, and all visible plant roots were handpicked for root mass measurement. The freeze‐dried soil samples were analyzed for other soil properties (e.g., microbial biomass) after homogenization and removal of stones and visible roots.

Gravimetric water content was determined by drying ∼5 g of fresh soil at 105°C for 48 h. Bulk density samples were dried at 105°C to a constant weight, weighed, and then subjected to the calculation of the ratio of the oven‐dried soil mass to the steel cylinder's volume (100 cm^3^). pH was measured at a soil:water ratio of 1:15 (w:v). SOC and TN of bulk soils were analyzed by an elemental analyzer (Vario EL III, Elementar, Hanau, Germany) after fumigation with concentrated HCl for 96 h to remove inorganic carbon. For measurement of Fe(II)_HCl_, fresh soils (∼1 g) were immediately soaked in 10 mL 0.5 M HCl for 12 h and shaken at 120 rpm for 1 h on a rotary shaker, and centrifuged at 10 000 g for 10 min, with the supernatant measured using the ferrozine absorbance method [58]. Specifically, Fe(II)_HCl_ was determined by absorbance at 562 nm on an UV and visible spectrometer (HACH DR1900) after mixing with 5 mM ferrozine solution. Notably, soil redox potential was measured in situ using a platinum electrode and a silver/silver chloride reference electrode in August 2023 and 2024.

Fourier transform infrared (FT–IR) spectroscopy. To further assess the degree of peat humification, we performed FT–IR spectroscopy analysis. Spectral characterization of peat samples was performed by diamond attenuated total reflectance FT–IR spectroscopy on a PerkinElmer Spectrum 3 FT–IR spectrometer fitted with a CsI beam splitter and a deuterated triglycine sulfate detector. An attenuated total reflectance (ATR) accessory made from a composite of zinc selenide and diamond, with a single reflectance system, was used to produce transmission‐like spectra. The freeze‐dried soil samples were ground to a fine powder. Samples were then placed directly on the crystal, and force was applied to ensure good contact between the crystal and the sample. Spectra were acquired by averaging 16 scans at 4‐cm^−1^ resolution (wavenumber) over the range 4,000–650 cm^−1^. The spectra were corrected for the ATR to allow for differences in depth of beam penetration at different wavelengths, and then baseline corrected with the instrument software (Figure S8). To calculate humification indices, we calculated ratios between absorbances at the following wavenumbers with respect to polysaccharides (1,030 cm^−1^): 2,920, 2,850, 1,630, and 1,515 cm^−1^ [59].

Overall, soil bulk density is less than 0.5 g cm^−3^ across the peat profiles (0–100 cm) in both waterlogged and drained sites (Figure S9), which is typical for peat profiles [60]. Compared to the waterlogged site, the drained site has higher bulk density (0.26 ± 0.01 vs. 0.23 ± 0.01 g cm^−3^) and lower soil water content (272 ± 16% vs. 309 ± 11%) in the top 50 cm(all *p* < 0.05; Figure S9), but there is no significant difference from 50 to 100 cm. Moreover, no significant differences were found between the two sites in terms of pH (5.15–5.83), SOC (28.6 ± 1.0% vs. 30.2 ± 1.1% for waterlogged and drained sites), SOC/TN (16.9 ± 0.3 vs. 16.4 ± 0.3), or any of the three humification indices (2920/1030, 2850/1030, 1630/1030). These findings suggest that peat depth at both of our sites is at least 1 m, and that the two sites had comparable peat decomposition.

### 4.4 Porewater Sampling

Porewater was sampled 14 times during the growing seasons of 2020–2024 using ceramic‐cup tension samplers (22 mm diameter, 100 mm length, 0.22 µm pore size) installed at 10, 30, and 50 cm depths at both sites. Sampling campaigns followed specific temporal and weather‐related criteria: all collections were conducted on sunny, rainless days and were scheduled to avoid periods of rapid water‐table fluctuation. In 2021 and 2022, sampling was performed almost monthly from May to August (i.e., early to peak growing season). In 2020, 2023, and 2024, sampling was conducted only in the early (May) and peak (August) growing seasons. Soils were frozen between October and April at 0–50 cm and hence not sampled. Approximately 400 mL of porewater was collected into HCl‐washed and sample‐rinsed 100 mL and 500 mL glass bottles sequentially, which were connected by a 2‐mm outside diameter tube. As a result, the porewater flowed into the 500‐mL bottle once the 100‐mL bottle overflowed, minimizing CO_2_ degassing and the associated pH changes [61]. The collected porewater was then transported on ice in a cooler to the laboratory in the field of Zoige and placed in a refrigerator at 4°C. Porewater was then split into six aliquots: (1) 20 mL was acidified to pH 2 with HCl and refrigerated at 4°C prior to DOC and total dissolved nitrogen (TDN) analysis; (2) 20 mL was immediately analyzed for Fe^2+^ and Fe^3+^; (3) 50 mL was frozen at −20°C prior to inorganic nitrogen (IN), element and anion analysis; (4) 30 mL was refrigerated at 4°C for dissolved phenol analysis; (5) 60 mL was frozen at −20°C for DOM molecular composition analysis; (6) 60 mL was frozen at −20°C for radiocarbon (^14^C) analysis. Due to the high cost associated with the latter two analyses, composite samples (360 mL) were prepared by mixing 60‐mL aliquots from six replicates per treatment group, collected at each depth and each site.

### 4.5 Dissolved Organic Matter Analyses

Concentrations of DOC and TDN were determined using a Multi N/C 3100 analyzer (Analytik Jena, Germany). Ammonium (NH_4_‐N) and nitrate (NO_3_‐N) were measured colorimetrically using an Autoanalyzer‐3 (Bran & Luebbe, Germany), and dissolved organic nitrogen (DON) was calculated as the difference between TDN and IN (sum of NH_4_‐N and NO_3_‐N). The DOC/DON ratio was used as an indicator of DOM source, i.e., plant‐derived DOM had high DOC/DON ratios relative to microbially processed soil organic matter [13]. Dissolved phenols mainly derived from plants were determined using the Folin‐Ciocalteu method outlined in Box [62] at 750 nm by a Multi‐Mode Microplate Reader (Synergy Mx, BioTek Instruments Inc., USA). Dissolved phenols were not analyzed in 2020 and May 2022 due to instrument malfunction.

### 4.6 ^14^C Analyses

To prepare DOC samples for ^14^C analysis, composite porewater was pooled from six replicates per treatment, depth, and site during the early (May) and peak (August) growing seasons in 2020–2022 and 2024. Due to insufficient sample volume, DOC from the 50 cm depth in the warmed plot of the waterlogged site was not analyzed for ^14^C in August 2022. The porewater and soil samples (collected before warming) were freeze‐dried, with inorganic carbon removed by fumigation with concentrated HCl for 96 h (no inorganic carbon was detected afterward). The acid‐treated samples were then analyzed for ^14^C via CO_2_ gas by Accelerator Mass Spectrometer (MICADAS, Ionplus AG, Switzerland) interfaced to an Elemental Analyzer (Elementar vario ISOTOPE select) [63], with CO_2_ gas directly injected into the ion source of MICADAS through the Gas Interface System (GIS) at the Ocean University of China radioCarbon Accelerator Mass Spectrometer Center (OUC‐CAMS). All ^14^C measurements were reported as F_m_ values, with conventional ^14^C ages (yr BP) calculated using the Libby half‐life following Stuiver and Polach [52]. Samples were normalized using oxalic acid II (NIST SRM4990C) and phthalic anhydride as a blank. The F_m_ precision of CO_2_ gas measurements for the international standard oxalic acid II was better than ±1%, whereas for phthalic anhydride, the F_m_ precision was less than 10%, and the F_m_ value was less than 0.005.

### 4.7 FT‐ICR MS Analysis

To examine DOM molecular composition, porewater collected in May and August 2020–2022 was analyzed by FT‐ICR MS. A known volume (100–200 mL) of composite porewater (pH 2.0) containing DOC of ∼3.0 mg was loaded onto a styrene divinyl benzene polymer solid phase extraction cartridge (Agilent, USA; Bond Elut PPL; 1 g) at a flow rate of 10 mL min^−1^ [64]. The loaded cartridges were rinsed with 5 mL of Milli‐Q water (acidified to pH 2.0 with HCl) three times to remove inorganics and dried under nitrogen gas for 30 min. Extracts were eluted with 8 mL of methanol and stored at −20°C until analysis on a 9.4‐Tesla Bruker Apex‐Ultra FT‐ICR MS equipped with an electrospray ionization (ESI) source. A diluted solution of the Suwannee River natural organic matter standard was used for peak calibration. Only peaks with a signal‐to‐noise ratio of >6 were considered for further analysis. Different parameters, including H/C ratio, AI_mod_ [39], NOSC [41], and double bond equivalents per carbon atom (DBE/C) [39], were calculated to reveal the molecular characteristics of DOM. NOSC was used to assess both the thermodynamic limitations on DOC decomposition and potential DOC sources [41, 65], with lipid‐like compounds showing the lowest values [41].

Considering the low level of phosphorus detected in DOM samples, the formula assignment of possible peaks was constrained as C_1‐80_H_2‐200_O_0‐40_N_0‐3_S_0‐2_, and the AI_mod_, NOSC, and DBE/C can be expressed as [39, 41]:

(1) AImod=(1+C−0.5O−S−0.5H)/(C−0.5O−S−N)

(2) NOSC=4−(4C+H−2O−3N−2S)/C

(3) DBE/C=(1+C−0.5H+0.5N)/C

where C, H, O, N, and S refer to the stoichiometric number of each respective element provided by FT‐ICR MS analysis. In addition, aliphatic molecules, commonly originating from microbial biomass in aquatic DOM, were classified as DBE/C < 0.3 and H/C ratio ≥ 1 [40]. All of the above parameters were estimated as the average of all formulas weighted by their relative intensity.

### 4.8 Porewater Chemistry Analyses

To assess the impact of Fe reduction on DOC release, Fe^2+^ concentration in the porewater was analyzed with ferrozine as previously described. Total Fe was first reduced using hydroxylamine hydrochloride (2%) and then analyzed as Fe^2+^ as above. Fe^3+^ was calculated as the difference between total Fe and Fe^2+^. Fe^2+^ and total Fe were analyzed within 24 h after water collection. Major cations, including Mn^2+^, Ca^2+^, Na^+,^ and Mg^2+,^ were determined by inductively coupled plasma optical emission spectroscopy (ICP‐OES, iCAP 6300, Thermo Scientific). Consistent with previous studies [66, 67], we operationally defined dissolved Mn as Mn^2+^, given that Mn^2+^ constitutes > 80% of total dissolved Mn under anaerobic conditions. Cl^−^, a conservative tracer to assess the effect of evapotranspiration on DOC concentration [33, 38], was measured using an ion chromatograph (ICS‐1500, Thermo Scientific Dionex) from most samples (except for May 2020, May 2021, and August 2024).

### 4.9 Microbial Biomass and Extracellular Enzyme Activity Analyses

Microbial biomass was determined by phospholipid fatty acids (PLFAs) using a modified Bligh‐Dyer method [68]. Briefly, ∼3 g of freeze‐dried soil was extracted in a mixture of dichloromethane, methanol, and 0.15‐M citrate buffer. Total phospholipids were separated from neutral lipids and glycolipids through a silica gel column. Both phospholipids and neutral lipids were converted into fatty acid methyl esters by alkaline methanolysis [69] and recovered with a hexane and dichloromethane mixture (4:1, v/v) three times. The extract was spiked with cholestane as an internal standard and evaporated to dryness under nitrogen gas. PLFAs were analyzed on a Trace 1310 gas chromatograph (GC) coupled to an ISQ mass spectrometer (MS; Thermo Fisher Scientific, USA) using a DB‐5MS column (30 m × 0.25 mm i.d., film thickness, 0.25 µm) for separation as described by Zhang et al. [70]. Microbial biomass is represented by total PLFAs [71], including all identified PLFAs (C_14_–C_19_, 20:0).

The activities of four extracellular hydrolases, β‐1,4‐glucosidase (BG), β‐N‐acetyl‐glucosaminidase (NAG), leucine aminopeptidase (LAP), and phosphatase (AP), were analyzed using fluorescence measurements [72], and phenol oxidase activity was assayed using spectrophotometry according to Saiya‐Cork et al. [73]. Conventional buffers were not used because we wanted to perform the assays at field pH rather than at optimal pH and ionic strength, as explained previously by Zhao et al. [28]. In brief, ∼1 g fresh soil was added to 45 mL Milli‐Q water and homogenized with a magnetic stirrer for 1 min. The resulting suspension (200 µL) was dispensed into 96‐well microplates (six replicate wells per sample per assay) with specific substrates (4‐methylumbelliferone (MUB)‐β‐D‐glucoside for BG, 4‐MUB‐N‐acetyl‐β‐D‐glucosaminide for NAG, L‐leucine‐7‐amino‐4‐methylcoumarin for LAP and 4‐MUB phosphate for AP) for four extracellular hydrolases or L‐3,4‐dihydroxyphenylalanine (L‐DOPA; 5 mM) for phenol oxidase added to each sample well, respectively. The resulting microplates were incubated in the dark at 20°C for 4 h for extracellular hydrolases and 3 h for phenol oxidase. Fluorescence was measured with excitation at 365 nm and emission at 450 nm using a Multi‐Mode Microplate Reader (Synergy Mx, BioTek Instruments Inc., USA) while absorbance was measured at 450 nm. Enzyme activity was expressed in the units of µmol h^−1^ g^−1^ dry soil.

### 4.10 Microbial Sequencing

To explore the potential role of microbial community (i.e., Fe‐reducing bacteria) in mediating Fe reduction, soil samples collected in August 2023 (after four years of warming) were analyzed for high‐throughput sequencing. DNA was extracted from ∼0.25 g of soil samples stored at −80°C using the E.Z.N.A. Soil DNA Kit (Omega Bio‐tek, USA). The quality and concentration of extracted DNA were determined by 1.0% agarose gel electrophoresis and a NanoDrop2000 spectrophotometer (Thermo Scientific, USA; 260/280 and 260/230 nm ratios). The V4 hypervariable region of the prokaryotic (bacterial and archaeal) 16S rRNA gene was amplified using primers 515Fmod (5′‐GTGYCAGCMGCCGCGGTAA‐3′) and 806RmodR (5′‐GGACTACNVGGGTWTCTAAT‐3′) [74]. The 20 µL polymerase chain reaction (PCR) mixture consisted of 10 µL 2 × Pro Tap, 0.8 µL each primer (5 µM), 1 µL of template DNA (10 ng µL^−1^), and ddH_2_O to volume. The thermal cycling protocol was as follows: initial denaturation at 95°C for 3 min, followed by 29 cycles of denaturing at 95°C for 30 s, annealing at 53°C for 30 s and extension at 72°C for 45 s min, then a single extension at 72°C for 10 min and ending at 10°C.

We used 16S rRNA gene primers targeting the same 515Fmod/806RmodR region of the 16S rRNA gene for the quantitative PCR (qPCR) analyses as used for the sequencing described above. Each qPCR reaction comprised of 10 µL 2 × Tap Plux Master Mix, 0.8 µL each primer (5 µM), 1 µL of template DNA, and ddH_2_O to volume, with the following thermal conditions: 95°C for 5 min, followed by 35 cycles of denaturing at 95°C for 30 s, annealing at 58°C for 30 s and extension at 72°C for 1 min.

Operational Taxonomic Units (OTUs) were clustered with a 97% similarity cut‐off using the Uparse software (Version 7.0.1090). Fe‐reducing bacteria (including *Bacteroides*, *Bacillus*, *Anaeromyxobacter*, *Geobacter*, *Desulfovibrio*, and *Pseudomonas*) were identified by matching genus‐level taxonomy in our samples to a literature‐derived Fe‐reducing bacteria database [49]. We acknowledge that the identification of Fe‐reducing bacteria based on 16S rRNA gene sequencing at the genus level does not directly confirm their Fe‐reducing functional activity, as not all species within the genera identified are capable of Fe reduction. Therefore, our results reflect the relative abundance of taxonomically defined putative Fe‐reducing bacteria rather than direct measurements of Fe‐reducing metabolic potential. The absolute abundance of Fe‐reducing bacteria was calculated by multiplying their relative OTU abundance by the qPCR‐derived mean genome copy number and expressed as copies per gram of dry soil (copies g^−1^ dry soil).

### 4.11 Radiocarbon Age of DOC Released by Microbial Fe Reduction

To estimate the age of DOC released via Fe reduction, a Bayesian hierarchical mixing model (Stan) was implemented via the “*rstan*” R package.

#### 4.11.1 Physical Constraint

Assuming the radiocarbon composition (F_m_) of the background (control) DOC pool remained unchanged under warming, the Fm value of the released DOC (*F_m_aged_*) was estimated based on an isotope mass balance:

(4) $$\begin{array}{ccc} & & F_{m\_warmed\_pred}\\ & & \;=\left(\mathrm{DO}C_{control}\times F_{m\_control}+\Delta DOC\times F_{m\_aged}\right)/\mathrm{DO}C_{warmed} \end{array}$$

The likelihood function links the model prediction to the observed F_m_ value of the warmed treatment:

(5) $$\begin{array}{c} F_{m\_warmed}∼\;Normal\;\left(F_{m\_warmed\_pred},\;\sqrt{se_{F_{m\_warmed}}^{2}+\partial_{extra}^{2}}\right) \end{array}$$

where *DOC_warmed_* and *DOC_control_* are porewater DOC concentrations at 10 cm depth in warmed and control plots, respectively; Δ*DOC = DOC_warmed_ − DOC_control_*; $F_{m\_warmed\_pred}$ is the predicted F_m_ value of the warmed treatment; $F_{m\_warmed}$ and $F_{m\_control}$ are the observed F_m_ values of the warmed and control plots, respectively; and *σ_extra_* accounts for additional unexplained variability. Measured uncertainty was propagated by assuming each observed value was normally distributed around its latent true value with the reported standard error (SE).

#### 4.11.2 Hierarchical Structure

To allow *F_m_aged_* to vary by season and year, we decomposed it as:

(6) $$F_{m\_aged\left[i\right]}=\mu +\beta_{Aug}\times I_{Aug}+u_{year\left[k\right]}$$

where *µ* is the baseline *F_m_aged_* for May; *β_Aug_* is the fixed offset for August (with *I_Aug_* a dummy variable, *I_Aug_* = 1 for August and 0 for May); *u_year[k]_* ∼ *Normal* (0, *σ_year_*) are year‐specific random intercepts. A larger *σ_year_* indicates greater interannual variability. Each value of *F_m_aged_* was calculated and converted to a conventional radiocarbon age (yr BP) [52].

#### 4.11.3 Priors

Weakly informative priors were assigned: *µ* ∼ *Uniform* (0, 1), *β_Aug_* ∼ *Normal* (0, 0.15), and *σ_year_*, *σ_extra_* ∼ *Half‐Cauchy* (0, 0.1). Prior sensitivity analysis confirmed results were data‐driven.

Posterior sampling used four chains, each with 2,000 warm‐up and 2,000 sampling iterations. Convergence was verified by *Ȓ* < 1.01, effective sample size > 400 for all parameters, and no divergent transitions. Results are reported as posterior means with 95% credible intervals. Posterior predictive checks indicated adequate model fit, with observed values falling within the 95% predictive intervals for the majority of data points.

### 4.12 Statistical Analysis

All statistical analyses were conducted using R version 4.3.2 (R Development Core Team, 2022). To present the temporal patterns clearly, we averaged the daily air temperature data from the data logger (recorded once every 30 min, 48 records per day, Figure S1). Shapiro‐Wilk and Levene's tests were used to check the normality and homogeneity of variance of the data, respectively. Two‐way analysis of variance (ANOVA) with repeated measures was used to test the effects of warming treatment and time on porewater parameters (e.g., DOC and Fe^2+^) at different depths (10, 30, and 50 cm) and aboveground biomass over five years (2020–2024). To meet the assumption of homogeneity of variances, data that violated this assumption were ln‐transformed prior to analysis. The analysis was performed using the *aov* function in R, followed by the Bonferroni test to assess the effect of warming over time. The linear mixed‐effects models were used to estimate the response of soil properties (e.g., Fe(II)_HCl_ and Fe‐reducing bacteria abundance) and root mass to warming over time, with the full factorial of warming treatment, soil depth, and time as fixed factors, and a random intercept for plot number. The linear mixed‐effects models (lme) were fitted by the *lme* function of the “*nlme*” R package. A non‐parametric Mann‐Whitney U test was used to determine the effects of warming treatments on DOM molecular composition (e.g., NOSC and AI_mod_) and DOC‐F_m_ values. It was also used to estimate the warming effects on soil properties at different soil depths. To identify the factors influencing DOC concentration, we assessed its relationships with aboveground biomass and major cations (e.g., Fe^2+^ and Ca^2+^) using Spearman correlation analysis. These data were ln‐transformed before analysis to achieve normal distribution. Additionally, we examined correlations between aboveground biomass and DOM composition indicators (e.g., dissolved phenols and AI_mod_), as well as between Fe^2+^ and DOC‐F_m_ values, using Spearman correlation on non‐transformed data. Differences and correlations were considered to be significant at *p* < 0.05.

## Author Contributions

X.F. conceptualized the study and supervised the project. G.D. and Z.L. established the warming experimental platform. G.D. conducted porewater, plant, and soil sampling and analyses with the assistance of Z.Y., W.H., L.M., E.K., W.L., Y.Z, C.L., J.J., and T.L. J.L., Y.G., and H.C. provided the field site and laboratory facilities in Zoige. H.Z. and M.Z. performed ^14^C‐DOC analyses. G.D. and X.F. analyzed the data and wrote the manuscript with contributions from all co‐authors.

## Conflicts of Interest

The authors declare no conflicts of interest.

## Supporting information




**Supporting File 1**: 76727‐sup‐0001‐SuppMat.docx.

**Supporting File 2**: advs76727‐sup‐0002‐DataSet.xlsx.

## Data Availability

The data that supports the findings of this study are available in the supplementary material of this article.
