# Supplementary material for: Iron‐Mediated Release of Aged Dissolved Organic Carbon From Waterlogged Peatland Under Warming
Source: Adv Sci (Weinh). 2026 Jul 23;13(57):e22234. doi: 10.1002/advs.202522234 (PMC13395398; doi:10.1002/advs.202522234)
Supplement: Supplementary file 1 [file ADVS-13-e22234-s001.docx]

**Supplementary Information**

**Iron-mediated release of aged dissolved organic carbon from waterlogged peatland under warming**

*Guohua Dai^1,2^, Zhiying Yang^1,2,3^, Zongguang Liu^1,2^, Wanjia Hu^1,2,3^, Lixiao Ma^1,2,3^, Enze Kang^1,2,3^, Wanqing Luo^1,2,3^, Yunpeng Zhao^1,2,3^, Chengzhu Liu^1,2,3^, Juan Jia^1,2^, Ting Liu^1,2^, Chen He^4^, Quan Shi^4^, Jianliang Liu^5,6^, Yongheng Gao^5,6^, Huai Chen^5,6,7^, Hailong Zhang^8^, Meixun Zhao^8^, Xiaojuan Feng^1,2,3*^*

*^1^State Key Laboratory of Forage Breeding-by-Design and Utilization, and Key Laboratory of Vegetation and Environmental Change, Institute of Botany, Chinese Academy of Sciences, Beijing 100093, China*

*^2^China National Botanical Garden, Beijing 100093, China*

*^3^University of Chinese Academy of Sciences, Beijing 100049, China*

*^4^State Key Laboratory of Heavy Oil Processing, China University of Petroleum, Beijing 102249, China*

*^5^Mountain Ecological Restoration and Biodiversity Conservation Key Laboratory of Sichuan Province,* *Chengdu Institute of Biology, Chinese Academy of Sciences, Chengdu 610213, China*

*^6^Zoige Wetland Ecology Research Station, Chengdu Institute of Biology, Chinese Academy of Sciences, Hongyuan 624400, China*

^7^Aba Teachers College, Aba 623002, China

*^8^Laoshan Laboratory, Qingdao 266237, China*

**Corresponding author: Xiaojuan Feng.*

*Email:* [*xfeng@ibcas.ac.cn*](mailto:xfeng@ibcas.ac.cn)

**This Supplementary Information contains:**

Figures S1 to S9

Figure S1. Location of Zoige peatlands, photos of experimental warming and control plots, and temperature effects. a location of Zoige peatlands on the northeastern Qinghai-Tibetan Plateau (QTP); the yellow line delineates the extent of QTP and the base map is derived from Esri World Imagery (<https://www.arcgis.com/home/item.html?id=10df2279f9684e4a9f6a7f08febac2a9>); b aerial photo of the study platform in waterlogged peatlands; c photo the study platform in drained peatlands; d photo of a warming plot; e four key methodological approaches used to quantify dissolved organic carbon (DOC) concentration, composition and sources and Fe-reduction process; f daily mean air temperature in warmed and control plots over five years (from August 2020 to August 2024). Different colors mean different treatments (blue indicates control and red indicates warming).

Figure S2. Water-table dynamics under control and warming treatments across growing seasons. Water-table position (cm) is shown for the waterlogged and drained regimes under control and warming treatments. Error bars represent standard error of water table (*n* = 6). For the waterlogged site, this site typically has standing water throughout the growing season. Consequently, we did not routinely measure the water-table. However, we observed a drought event in 2023, which prompted us to begin measuring water levels at this site.

Figure S3. Temporal changes in soil temperature at different depths under warming relative to the control over five years (from August 2019 to August 2024). Warming-induced soil temperature change (ΔT, °C; warming − control) is shown for waterlogged and drained peatlands at 10, 30 and 50 cm depths. a waterlogged site; b drained site. Grey points denote daily mean ΔT, and the red line shows the 7-day rolling mean. Insets report the mean warming effect (mean ΔT ± s.e.). Grey shaded intervals indicate periods with missing data.

Figure S4. Effects of warming on aboveground biomass and root mass at drained and waterlogged sites. a, b drained site; c, d waterlogged site. + and − show significant increase and decrease (*p* < 0.05), respectively. Error bars are standard errors of the mean values (*n* = 6).

Figure S5. Effects of warming on soil microbial biomass (indicated by PLFAs) and extracellular enzyme activities (NAG, BG, AP, LAP, PO) after two (2021) and four years (2023) of warming treatment at drained and waterlogged sites. a drained site; b waterlogged site. Note: microbial biomass data are shown for the pre-treatment period (2019) and after two years of warming (2021). + and − show significant increase and decrease (*p* < 0.05), respectively. Error bars are standard errors of the mean values (*n* = 6). PLFAs: phospholipid fatty acids; NAG: β-N-acetyl-glucosaminidase; BG: β-1,4-glucosidase; AP: phosphatase; LAP: leucine aminopeptidase; PO: phenol oxidative.

Figure S6. Ratios of dissolved organic carbon to chloride (DOC/Cl^−^) at 10 cm depth from control and warmed plots at drained and waterlogged sites. Solid lines in violins mark the mean value of each dataset. Error bars are standard errors of the mean values (*n* = 66).

Figure S7. Effects of warming on soil redox potential after four (2023) and five years (2024) of warming treatment at drained and waterlogged sites. a waterlogged site; b drained site. + and − show significant increase and decrease (*p* < 0.05), respectively. Error bars are standard errors of the mean values (*n* =6).

Figure S8. Average peat FT-IR absorption spectra at 0‒10, 20‒30 and 40‒50 cm depths, with standard errors (*n* = 3) as shaded areas. Spectra are stacked (i.e., absorbance = 0 at each apparent baseline) and sized to the same vertical scale.

Figure S9. Soil properties at waterlogged and drained sites. a water content and bulk density across the soil profile (0‒100 cm); b-i soil organic carbon (SOC), SOC to total nitrogen (TN) ratio (SOC/TN), pH, and FT-IR derived humification indices (ratios of absorption at wavenumbers 2920/1030, 2850/1030, and 1630/1030) at 0‒10, 20‒30 and 40‒50 cm depths. Error bars are standard errors of the mean values (*n* = 3).
